# Angiotensinogen Variants among Resistant Hypertensive Patients

**DOI:** 10.1155/2014/424793

**Published:** 2014-03-24

**Authors:** Natalia Ruggeri Barbaro, Vanessa Fontana, Heitor Moreno

**Affiliations:** Laboratory of Cardiovascular Pharmacology, Faculty of Medical Sciences, University of Campinas, FCM 10 Building, 1st Floor, 13083-970 Campinas, SP, Brazil

The recently published paper entitled “*Genetic and adverse health outcome associations with treatment resistant hypertension in GenHAT*” by Lynch et al. [1] evaluated the association between 78 candidate gene polymorphisms and treatment resistant hypertension (TRH). Interestingly, the main finding was the association of two genetic variants in the angiotensinogen (*AGT*) gene, the M allele of rs699 and the G allele of rs5051, and TRH in white but not in African-American subjects. Previous Brazilian studies [2, 3] found the association with this SNP but at an opposite direction; carriers of the* AGT *M235T T allele, not Met allele, were at increased risk for resistant hypertension. The divergence among those studies might be explained by the differences between the genetic structure of the studied populations. The high degree of miscegenation among Brazilians, including Africans, Europeans, and Ameridian ancestral roots, is well known [4, 5]. Admixed populations have a distinct pattern of linkage disequilibrium that affects haplotype blocks and might explain those apparent divergent findings. Also, it is important to note that* AGT* M235T polymorphism reported in those studies might not be the causal variant, and the causation cannot be drawn. Moreover, the present clinical study showed evidence of a significant interaction by race in the association of* AGT* M235T rs699 with TRH, the Met allele being less frequent among African-American than white participants, highlighting the importance of the race in resistant hypertension susceptibility. Undoubtedly, the present study has great contribution in the genetic background of resistant hypertension and contributes to improvement of the knowledge in such condition.
